# GNG5 Controls the Number of Apical and Basal Progenitors and Alters Neuronal Migration During Cortical Development

**DOI:** 10.3389/fmolb.2020.578137

**Published:** 2020-11-02

**Authors:** Ane Cristina Ayo-Martin, Christina Kyrousi, Rossella Di Giaimo, Silvia Cappello

**Affiliations:** ^1^Max Planck Institute of Psychiatry, Munich, Germany; ^2^International Max Planck Research School for Translational Psychiatry (IMPRS-TP), Munich, Germany; ^3^Department of Biology, University of Naples Federico II, Naples, Italy

**Keywords:** GNG5, human cortical development, basal progenitor cells, neuronal migration, cerebral organoids

## Abstract

Cortical development is a very complex process in which any temporal or spatial alterations can give rise to a wide range of cortical malformations. Among those malformations, periventricular heterotopia (PH) is characterized by clusters of neurons that do not migrate to the correct place. Cerebral organoids derived from patients with mutations in *DCHS1* and *FAT4*, which have been associated with PH, exhibit higher levels of *GNG5* expression in a patient-specific cluster of neurons. Here we investigate the role of GNG5 during the development of the cerebral cortex in mice and human cerebral organoids. *GNG5*, highly expressed in progenitors and downregulated in neurons, is critical for controlling the number of apical and basal progenitors and neuronal migration. Moreover, forced expression of *GNG5* recapitulates some of the alterations observed upon downregulation of *Dchs1* and *Fat4* in mice and human cerebral organoids derived from *DCHS1* and *FAT4* patients, suggesting a critical role of *GNG5* in cortical development.

## Introduction

The development of the cerebral cortex is a very sophisticated process, and any disturbances may lead to cellular defects that can result in a range of cortical malformations. For instance, alterations in the normal generation or function of neural progenitors and neurons often result in neuronal migration disorders (Buchsbaum and Cappello, 2019). Recent studies have demonstrated that mutations in the protocadherin genes *DCHS1* and *FAT4* are associated with Van Maldergem Syndrome (Cappello et al., 2013). One of the central features of patients affected by Van Maldergem Syndrome is the presence of gray matter in the periventricular area. Periventricular heterotopia (PH) is a disorder characterized by the presence of ectopic neurons at the lateral ventricles and often associated with seizures (Barkovich and Kuzniecky, 2000; Aghakhani et al., 2005; Mansour et al., 2012; Rispoli et al., 2013).

Recent data achieved by modeling PH in human cerebral organoids (COs) derived from patients with mutations in *DCHS1* and *FAT4* revealed the molecular and cellular mechanisms involved in the formation of PH. At the progenitors’ level, both DCHS1 and FAT4 are critically involved in the maintenance of the correct number and polarity of apical radial glial cells (aRGs). Besides, they are essential in establishing neuronal migration dynamics as shown by live imaging. Transcriptome analysis allowed the identification of a specific cluster of neurons with an altered neuronal state (Klaus et al., 2019). Given that PH is a disorder that results in migration defects of only a subset of neurons, while the majority of neurons reach the correct destination, it is now possible to tackle which set of dysregulated genes may be responsible for the formations of bands or nodules accumulating at the ventricles and to propose strategies that could affect only altered cells. Among the differentially regulated genes in the altered neuronal cluster, *GNG5* was the most dysregulated gene (Klaus et al., 2019). *GNG5* is naturally expressed in progenitors and particularly in human basal radial glial cells (bRGs) but downregulated in neurons in both mice and humans (Pollen et al., 2016; Kanton et al., 2019; Telley et al., 2019). However, altered mutant neurons keep a high expression of *GNG5*, suggesting that its downregulation is essential for proper migration (Klaus et al., 2019).

*GNG5* encodes for the G protein subunit gamma 5 (Gγ5). Together with α and β subunits, they form a heterotrimeric protein complex, that interacts with the G protein-coupled receptors (GPCRs) on the membrane and transduce the signal intracellularly (Gilman, 1987; McCudden et al., 2005). Binding of an agonist to the GPCRs induces the release of the α subunit due to the exchange of GDP to GTP and the release of the βγ subunits allowing them to be the effectors in a wide range of pathways (MAP Kinase cascade or vesicular traffic among others) (Clapham and Neer, 1993, 1997; Wedegaertner et al., 1995; Hewavitharana and Wedegaertner, 2012).

*Gng5* knockout mice die at embryonic day 10.5 due to heart and head defects (Moon et al., 2014). Moreover, *Gng5* transcripts are highly enriched in mouse neural progenitor cells in both embryonic and adult brain (Morishita et al., 1999; Asano et al., 2001; Telley et al., 2019). In the developing human brain and human-derived COs, it is highly expressed in bRGs (Pollen et al., 2016; Kanton et al., 2019; Polioudakis et al., 2019), a type of progenitor cells enriched in gyrified species, where they have a role in brain expansion and gyrification (Fietz et al., 2010; Kelava et al., 2012; Florio and Huttner, 2014; Penisson et al., 2019). Taken together, we hypothesized that GNG5 might play a crucial role in the development of the human brain.

Using a combination of *in vitro* induced pluripotent stem cell (iPSC) derived COs and *in vivo* mouse models, we shed new light on the role of GNG5 in neural progenitors and neurons during development.

## Materials and Methods

### Cloning

For the *pCAGGS-GFP-IRES-GNG5* plasmid, the open reading frame (ORF) of human *GNG5* was cloned into a *pCAGGS* (Cappello et al., 2013) plasmid following standard cloning methods. The primers to amplify *GNG5* from cDNA from neuroblastoma (SH-SY5Y) were the following:

– Forward: TCCTCTTCAGACCCCCTCTT
– Reverse: ATTGTATGCTGCTGCCAGTG

The primers to amplify the ORF and introduce the restriction sites to clone the gene in the vector were the following:

– Forward with NheI RS: AAAGCTAGCATGTCTGGCTC CTCCAGC
– Reverse with *EcoRV* RS: ATAGATATCCTACAAAAAGGA ACAGACTTTCTGGGG

The *pCAGGS-GFP-IRES-GNG5* overexpression plasmid as well as the empty *pCAGGS-GFP*, used as a control, were electroporated in COs and mice.

For the analysis of the cell profile, COs were co-electroporated with a *pCAGGS-GAP43-GFP* plasmid (Cappello et al., 2006; Attardo et al., 2008; Klaus et al., 2019) while in mice the *pCAGGS-GFP-IRES-GNG5* plasmid was co-electroporated with the control *pCAGGS-GFP*.

For the intracellular localization of GNG5, a *CMV-GNG5-GFP* tagged plasmid was purchased from GenScript (GNG5_ OHu09122C_*pcDNA3.1*(+)*-eGFP*, Clone ID:OHu09122C, ORF NM_005274.3).

### Plasmid Preparation

The preparation of small-scale plasmid was obtained with the QIAprep Spin Miniprep Kit (Qiagen) and the large-scale preparation with the Endofree Plasmid Maxi Kit (Qiagen) after the transformation in Subcloning Efficiency^TM^ DH5α^TM^ Competent Cells (Thermo Fisher Scientific).

### iPSC Culture

HPS0076 cells (Okita et al., 2011) were obtained from the RIKEN Bioresource Center, Japan. Cells were kept at 37°C, 5% CO_2_ and ambient oxygen level. They were cultured in Matrigel^®^ Basement Membrane Matrix, LDEV-free (354234, Corning^®^) coated plates in mTESR1 medium supplemented with 1× mTESR1 supplement (85850, Stem Cell Technologies). Media was changed every day. For passaging, iPSCs were washed once with Dulbecco’s Phosphate Buffered Saline (PBS) and kept in Accutase^®^ solution (A6964, Sigma Aldrich) diluted 1:4 in PBS for 5 min at 37°C. Floating colonies were washed with DMEM/F-12 + Glutamax and centrifuged at 300 g for 5 min. Collected colonies were resuspended in mTESR1 with 1× mTESR1 supplement and 10 μM Rock inhibitor Y-27632(2HCl) (72304, Stem Cell Technologies) and diluted in the desired density.

### Cerebral Organoids Generation

The generation of COs was performed as described previously (Lancaster and Knoblich, 2014; Klaus et al., 2019; Penna et al., 2019). In short, iPSCs were washed in PBS and treated with Accutase^®^ solution for 5 min at 37°C to get single cells. 9000 cells/well were plated in Round Bottom Ultra-Low Attachment 96-well plates containing *hES medium* [DMEM/F12+Glutamax, 20% of KnockOut^TM^ Serum Replacement, 3% of hESC-quality Fetal Bovine Serum (FBS), 0.1 mM of 2-mercaptoethanol (50 mM), 1% of MEM Non-Essential Amino Acids Solution (100×), 4 ng/ml basic fibroblast growth factor (bFGF/FGF-2) and 50 μM Rock inhibitor Y-27632(2HCl)]. Cells were kept in *hES medium* to generate Embryoid Bodies (EBs). Rock inhibitor and bFGF were removed after day 4. After day 6 EBs were transferred to Ultra-Low Attachment 24-well plates containing *NIM medium* (DMEM/F12+Glutamax supplemented with 1:100 N2^TM^-Supplement (100×), 1% of MEM Non-Essential Amino Acids Solution and 5 μg/ml heparin) and kept in culture for another 6 days. On day 12, EBs were embedded in Matrigel^®^ Basement Membrane Matrix and moved to 10 cm dishes (30 EBs per plate) containing *NDM-A medium* (DMEM/F12+Glutamax and Neurobasal^TM^ Medium in a ratio 1:1 with 1:200 N2^TM^-Supplement (100×), 1:100 B-27^TM^ Supplement (50×) minus vitamin A, 0.5% of MEM Non-Essential Amino Acids Solution (100×), 0.5% GlutaMAX^TM^ Supplement, 50 μM of 2-mercaptoethanol (50 mM), antibiotic antimycotic Solution (100×) and insulin 2.5 ug/ml). 4 days after keeping the COs in *NDM-A medium* they were transferred to *NDM+A medium* (DMEM/F12+Glutamax and Neurobasal^TM^ medium in a ratio 1:1 supplemented with 1:200 N2^TM^-supplement (100×), 1:100 B-27^TM^ supplement (50×), 0.5% of MEM Non-Essential Amino Acids Solution (100×), 0.5% GlutaMAX^TM^ Supplement, 50 μM of 2-mercaptoethanol (50 mM), antibiotic antimycotic Solution (100×) and Insulin 2.5 μg/ml and moved to an orbital shaker. Media was changed every three days. During the entire COs generation procedure cells were kept at 37°C, 5 % CO_2_ and ambient oxygen level.

### Generation of Neuronal Progenitor Cells

Neuronal Progenitor Cells (NPCs) were generated as previously described (Boyer et al., 2012; Klaus et al., 2019) with small modifications. In short, iPSC colonies were incubated for 15 min with Collagenase Type IV (7909; StemCell Technologies), colonies were washed with DMEM/F12 and detached by manual disruption. Detached colonies were grown in suspension in *NIM medium* (DMEM/F12 + HEPES with 1:200 N2^TM^-Supplement (100×) and 1:100 B-27^TM^ Supplement (50×) minus vitamin A) for the generation of EBs. Media was changed every other day. After 1 week in suspension, EBs were plated on polyornithine/laminin-coated plates. 1 week after, the neural rosettes grown from the plated EBs were picked and plated in a new polyornithine/laminin-coated plate. Resulting cells, considered NPCs, were cultured in *NPM medium* (*NIM medium* supplemented with 20 ng/ml bFGF/FGF-2) and passaged with Accutase^®^ with a maximum splitting ratio of 1:5. NPCs were only used for up to ten passages.

### Cell Lines

Human neuroblastoma cells (SH-SY5Y) were grown in DMEM/F12+Glutamax supplemented with 10% of FBS and 1% of Antibiotic Antimycotic Solution (100×). Cells were kept at 37°C, 5% CO_2_ and ambient oxygen level.

### Nucleofection of Neuroblastoma (SH-SY5Y) Cells

SH-SY5Y cells were kept in culture until they were 75-80% confluent. After washing with PBS cells were dissociated with 0.05% trypsin-EDTA until single cells were obtained. 5 million cells were resuspended in 500 μl of nucleofection buffer (50 mM Hepes, 90 mM Na_3_PO_4_, 5 mM KCl, 0.15 mM CaCl_2_) and 20 μg of the *CMV-GNG5-GFP* plasmid were added. The mix was added into an aluminum electrode cuvette and exposed to program G00.04 in the Amaxa II Nucleofector (Lonza). Sequentially, 5 million cells/15 cm dish were plated. Transfected cells were cultured for 48 h.

### Transfection of NPCs by Lipofection

NPCs were transfected with the *CMV-GNG5-GFP* plasmid using Lipofectamine^TM^ 3000 Transfection Reagent (L3000001, Thermo Fisher Scientific) following the manufacturer’s protocol.

### Electroporation of COs

COs were electroporated at two different time points: at day 20 and day 35 after plating the iPSCs on the 96-well plate. For electroporation, COs were kept in *NDM+A medium* without Antibiotic Antimycotic solution and moved to an electroporation chamber (Harvard Apparatus). Using a stereoscope to localize the ventricle-like cavity (VL), 1–2 μl of each plasmid (*pCAGGS-GFP*, *pCAGGS-GFP-IRES-GNG5*, 2/3 of *pCAGGS-GFP* + 1/3 of *pCAGGS-GAP43-GFP* or 2/3 of *pCAGGS-GFP-IRES-GNG5* + *1/3 of pCAGGS-GAP43-GFP*) to a final concentration of 1 μg/μl mixed with 0.1% Fast-Green (F7252, Sigma Aldrich) were injected using Glass Micropipettes (5-000-1001-X10, Drummond Scientific) and electroporated with five pulses applied at 80 mV for 50 ms each at intervals of 500 ms (ECM830, Harvard Apparatus). 24 hours after electroporation COs were moved to new *NDM+A medium* and kept in culture for 7 additional days until they were fixed for 2 h in 4% PFA. After fixation, COs were transferred to 30% sucrose in PBS overnight for cryopreservation, embedded in OCT Compound (361603E, VWR Chemicals) and stored at −20°C.

For immunohistochemistry, 14 μm sections were prepared with a cryostat. For each analysis, at least 3 different COs per condition were analyzed from 2 independent batches.

### *In utero* Electroporation in Mice

For *in utero* electroporation pregnant C57BL/6 mice were used under the license number 55.2-1-54-2532-79-2016 approved by the Government of Upper Bavaria. Animals were anesthetized by an intraperitoneal injection containing a saline solution with fentanyl (0.05 mg per kg body weight), midazolam (5 mg per kg body weight) and medetomidine (0.5 mg per kg body weight) (Btm license number 4518395). Embryos were electroporated at E13 following the protocol by (Saito, 2006).

1–2 μl of each plasmid (*pCAGGS-GFP*, *pCAGGS-GFP-IRES-GNG5*, 1/3 of *pCAGGS-GFP* + 2/3 of *pCAGGS-GFP-IRES-GNG5*, or *CMV-GFP-GNG5*) to a final concentration of 1 μg/μl and mixed with 0.1% Fast-Green (F7252, Sigma Aldrich) were injected using Glass Micropipettes (5-000-1001-X10, Drummond Scientific). After finalizing the electroporation, anesthesia was concluded by injection of buprenorphine (0.1 mg per kg body weight), atipamezole (2.5 mg per kg body weight) and flumazenil (0.5 mg per kg body weight). Brains were fixed 1 days post electroporation (dpe), 3 or 6 dpe in 4% PFA for 4 h (1 dpe) or overnight (3 and 6 dpe) and were subsequently transferred to 30% sucrose in PBS overnight for cryopreservation, embedded in OCT Compound afterward and stored at −20°C until further use.

For immunohistochemistry, 12 μm sections were arranged using a cryostat. For each analysis, at least 2 different mice brains per condition were analyzed.

### Immunostaining

Frozen mouse brain and CO sections were thawed for 20 min at room temperature (RT) and rehydrated with PBS for 5 min.

For nuclei antigen exposure, antigen retrieval was performed using fresh citric buffer (0.01 M, pH = 6) and the sections were incubated in it for 1 min at 720 W and 10 min at 120 W. Sections were cooled down for 20 min after which half of the citric buffer was exchanged with water and cooled down for another 10 min. Finally, sections were washed with PBS for 5 min and continued with the standard immunostaining protocol.

After, the PBS washed sections were fixed again with 4% PFA for 10 min. Sections were then washed twice with PBS and treated with 0.3% Triton in PBS for 5 min. Then, sections were washed 3 times with PBS for 5 min before blocking (10% Normal Goat Serum (S-1000, Vector Laboratories), 1% BSA in 0.1% Tween in PBS) for 1 h at RT.

After blocking, sections were incubated with primary antibody diluted in blocking solution at the desired concentration overnight at 4°C (antibody list on Table 1). Following several washes, sections were incubated with AlexaFluor-conjugated secondary antibodies (Life technologies) at 1:1000 dilution together with 0.1 μg/ml 4,6-diamidino-2-phenylindole (DAPI) (D9542, *Sigma Aldrich*) to detect nuclei. F-ACTIN was visualized by incubation with Alexa Fluor 594-conjugated PHALLOIDIN (A12381, Thermo Fisher Scientific) following the manufacturer’s protocol.

**TABLE 1:** Antibody list.

Antigen	Dilution	Vendor	Catalogue #
GFP	1:1000	Aves Lab	GFP-1020
PAX6	1:500	Millipore	ab2237
PH3	1:500	Millipore	06-570
TBR2	1:500	Abcam	ab23345
β-CATENIN	1:500	BD Biosciences	610154
LAMININ	1:500	Millipore	ab2034
CTIP2	1:500	Abcam	ab18465
SATB2	1:500	Abcam	ab51502
MAP2	1:500	Sigma Aldrich	M4403
NEUN	1:500	Millipore	MAB377
PHALLOIDIN (ACTIN)	1:40	Thermo Fisher	a12381
TOM20	1:500	Santa Cruz	SC-11415

SH-SY5Y and NPCs were grown in 24-well plates with coverslips and fixed for 15 min with 4% PFA at RT. Afterward, the immunostaining protocol was performed as for COs and mouse brain sections.

### Confocal Imaging

Frozen mouse brain and COs sections, as well as cells, were visualized through a Leica SP8 confocal laser-scanning microscope with 10× and 40× (water immersion) objectives.

### Image Analysis, Quantification and Statistical Analysis

For *in vivo* analysis after *in utero* electroporations, cell quantifications were carried out in Adobe Photoshop CS6. For the binning analysis, brain sections were divided into 5 equal bins. At least 2 sections were counted for 6 control (CTRL) and 4 *GNG5* OX brains at E13-E14 for GFP and 5 CTRL and 4 *GNG5* OX brains for Tbr2 and 4 CTRL and 7 *GNG5* OX brains at E13-E16. For the neuronal disruption phenotype, 6 CTRL and 8 *GNG5* OX brains at E13-E16 and 3 CTRL and 8 *GNG5* OX brains at E13-E19 were analyzed. The apical belt integrity was measured at E13-E16 in 6 CTRL and 8 *GNG5* OX. The tortuosity of the radial glial cells was obtained at E13-E14 in 2 CTRL and 2 *GNG5* OX in which 29 and 24 cells were analyzed, respectively. The tortuosity of the RG processes was assessed by measuring the real length of the RG process and dividing this value by the direct distance from the beginning to the end of the process.

For the analysis of electroporated COs, cell quantification was carried out using Fiji (Schindelin et al., 2012). Several COs were analyzed from 2 independent batches. Data are represented with *n* = number of analyzed VL cavities.

Neuronal intrusions were analyzed by MAP2^+^ staining. The VLs from different electroporated COs were included in the *processes* category when there were three or more MAP2+ processes inside the GZL of that electroporated VL and in the *cell bodies* category whenever there was one MAP2+ cell body in the apical site of the GZL or more than two MAP2+ cell bodies in the GZL.

The presence of neuronal cell bodies was further assessed by the presence of ectopic NEUN+ nuclei in the GZL. The VLs from different electroporated COs were included in the *cell bodies* category whenever there was a NEUN+ cell body in the apical site of the GZL or more than two NEUN+ nuclei in the entire GZL.

The disruption of the apical belt was assessed by PHALLOIDIN staining in COs and β-CATENIN in mice. A VL or a brain in which the apical belt was altered were included in the *disrupted apical belt* category whenever there were areas without PHALLOIDIN staining surrounded by electroporated cells and with an intact DAPI staining.

Statistical analysis and data representation were performed with GraphPad Prism^®^ version 6.01. The statistical test performed for each analysis is stated in each figure legend.

## Results

### GNG5 Is Highly Expressed in Progenitor Cells and Localizes to Mitochondria

In mice, *Gng5* is highly expressed in progenitor cells from very early stages of development (Supplementary Figures 1A,B), while *Dchs1* and *Fat4* are enriched in neurons (Supplementary Figures 1A,C,D). In human *in vivo* and *in vitro* samples, *GNG5* is also highly expressed in progenitors from the beginning of neurogenesis, particularly in bRGs (Supplementary Figures 1E,F and 2A–C) whereas *DCHS1* and *FAT4* are expressed in some progenitors and some neurons with higher expression in neurons through development (Supplementary Figures 1E,G,H and 2A,B,D,E). These data suggest that *GNG5*, *DCHS1* and *FAT4* may have a complementary role. To investigate the cellular localization of GNG5 in absence of specific antibodies, we overexpressed a *CMV-GNG5-GFP* plasmid, where *GNG5* is fused with *GFP* and we monitored its localization in human neuroblastoma (SH-SY5Y) and human NPCs after transfection, and in mouse embryos electroporated at embryonic day 13 (E13) and analyzed 3 dpe. The results indicated that GNG5 colocalizes with TOM20 (Supplementary Figures 2F–H), a protein specifically found in mitochondria, indicating a clear enrichment of GNG5 in mitochondria.

### Forced Expression of *GNG5* Induces Premature Delamination and Neuronal Migration Defects in Human-Derived COs

**FIGURE 1:**
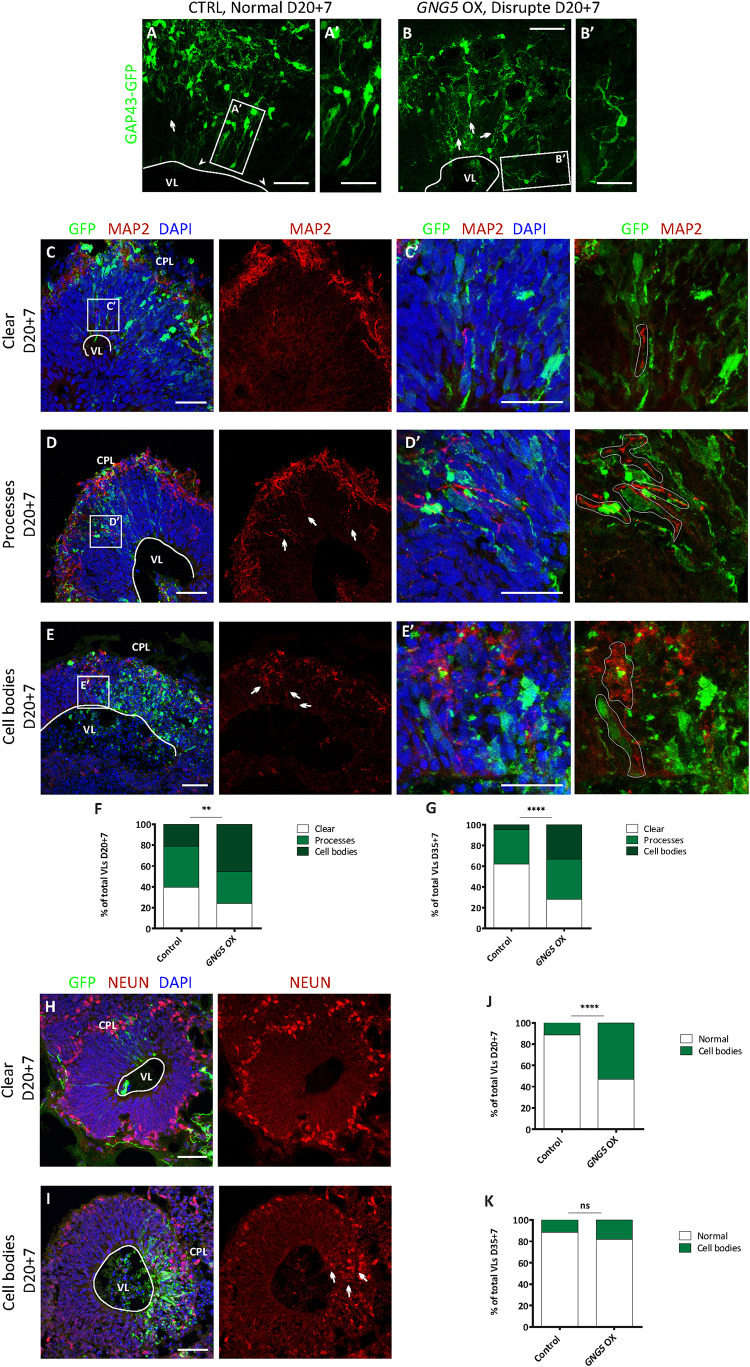
Acute overexpression of *GNG5* induces morphological changes and migration problems in COs. **(A–B′)** Representative images of VLs from COs co-electroporated with control or *GNG5* OX plasmid together with the *GAP43*-*GFP* plasmid. Arrowheads indicate intact radial glial cells, while arrows indicate disrupted or delaminated cells. **(C,E′)**. Representative pictures of MAP2 staining in COs after the electroporation of *GNG5* OX plasmid. **(C,C′)** Some of the VLs appear without any MAP2 staining in the GZL. **(D,D′)** Some VLs present processes from migrating neurons. **(E,E′)** In others, neurons are not able to migrate to their destination, and the neuronal cell bodies stay in the GZL. Arrows indicate the processes or cell bodies located in the GZL. **(F)** Percentage of VLs from control and *GNG5* OX COs without MAP2 staining in the GZL (clean), MAP2 processes or cell bodies at 20 + 7 and **(G)** at 35+7 days. (**H,I)**. Representative pictures of NEUN staining in COs after the electroporation of *GNG5* OX plasmid. **(H)** Some of the VLs appear without any NEUN+ nuclei in the GZL. **(I)** Some VLs present ectopic neurons in the GZL. Arrows indicate the cell bodies located in the GZL. **(J)** Percentage of VLs from control and *GNG5* OX COs without NEUN+ ectopic neurons in the GZL (clean) or NEUN+ cell bodies at 20 + 7 and **(K)** at 35+7 days. MAP2 statistical analysis was based on the multinomial Chi-Square goodness of fit test, and the NEUN statistical analysis was based on exact binomial test ^∗∗^*p* < 0.01, ^****^p < 0.0001. MAP2: 20 + 7 CTRL batches (b) = 2, COs (*o*) = 11, VLs (v) = 61; *GNG5* OX *b* = 2, *o* = 11, *v* = 33 and 35+7 CTRL *b* = 2, *o* = 8, *v* = 21; *GNG5* OX *b* = 2, o = 9, v = 18. NEUN: 20 + 7 CTRL *b* = 2, *o* = 9, *v* = 35; *GNG5* OX *b* = 2, *o* = 9, *v* = 17 and 35+7 CTRL *b* = 2, *o* = 7, *v* = 17; *GNG5* OX *b* = 2, *o* = 4, *v* = 11. Scale bar: **(A′,B′)** 20 μm, **(C′,D′,E′)** 30 μm and **(A–E,H,I)** 50 μm. Abbreviations: CPL, cortical plate-like area; CTRL, control; GZL, germinal zone-like area; OX, overexpression; VL, ventricle-like cavities.

**FIGURE 2:**
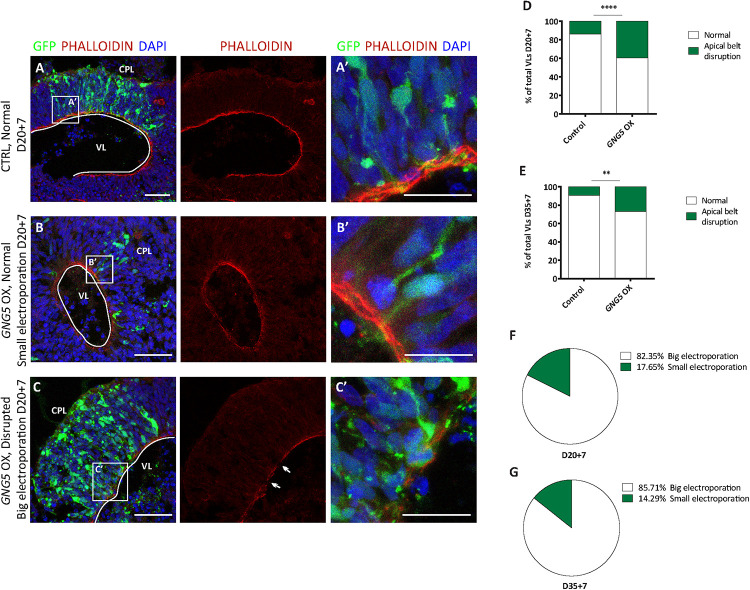
Acute overexpression of *GNG5* induces delamination problems in COs. **(A,A′)** Representative picture of an intact apical belt in control COs stained with the actin marker PHALLOIDIN, **(B,B′)** intact apical belt with small electroporation in *GNG5* OX COs and (**C,C′**) disrupted in *GNG5* OX COs with big electroporation. Arrows indicate the location of the disrupted belt. **(D)** Percentage of VLs with an intact or disrupted apical belt at 20 + 7 days and **(E)** 35+7 days. (**F**) Percentage of VLs with a small or big electroporation and a disrupted apical belt after GNG5 OX at 20 + 7 days and (**G**) 35+7 days. PHALLOIDIN statistical analysis was based on exact binomial test ^∗∗^*p* < 0.01, ^****^p < 0.0001. PHALLOIDIN: 20 + 7 CTRL *b* = 2, *o* = 11, *v* = 68; *GNG5* OX *b* = 2, *o* = 10, *v* = 43 and 35+7 CTRL *b* = 2, *o* = 5, *v* = 21; *GNG5* OX *b* = 2, *o* = 7, *v* = 26. Scale bar: **(A′,B′,C′)** 30 μm, and **(A–C)** 50 μm. Abbreviations: CPL, cortical plate-like area; CTRL, control; GZL, germinal zone-like area; OX, overexpression; VL, ventricle-like cavities.

To identify its role in early development and neurogenesis, we overexpressed *GNG5* by electroporation of a *pCAGGS-GNG5-IRES-GFP* plasmid into the VLs of COs at day 20, when most of the cells are progenitors, and at 35 days, a more mature stage with progenitors and neurons organized in clear germinal zone-like (GZL) and cortical plate-like (CPL) areas. COs were analyzed 7 dpe. To better highlight the cell morphology, COs were additionally co-electroporated with a plasmid coding for the membrane localization sequence of *GAP43* fused to *GFP* (*pCAGGS-GAP43-GFP*). This fusion protein directly localizes at the cell membrane highlighting the cell profile without interfering with the function of the wildtype protein (Cappello et al., 2006; Attardo et al., 2008; Klaus et al., 2019). At 20 + 7 days, COs overexpressing *GNG5* show a disrupted morphology, with less precise and straight processes in transfected progenitor cells (Figures 1A–B′). Previous reports have demonstrated that aberrant neuronal migration can be the consequence of defective morphology of radial glial cells (RGs) (Cappello et al., 2012, 2013; Klaus et al., 2019). Hence, we performed a detailed analysis of the position of neurons in the electroporated VL cavities by quantifying the presence of neuronal processes and neuronal cell bodies in the GZLs (Figures 1C–K). For that purpose, we used two different markers, MAP2 (Figures 1C–E′) for the identification of the neuronal outline and NEUN (Figures 1H,I) for the visualization of neuronal nuclei. Already at 20 + 7 days in culture, the number of VLs with MAP2+ neuronal process and cell bodies in the GZL were strongly increased upon overexpression of *GNG5* (*GNG5* OX) compared to COs electroporated with the control *GFP* plasmid (45% of the VLs with MAP2+ neuronal cell bodies in the GZL *in GNG5* OX and 21% in controls; 30% of the *GNG5* OX VLs with neuronal processes in the GZL and 39% in controls) (Figure 1F). The presence of ectopic neuronal nuclei in the GZL was also identified by NEUN staining at this stage (52% of the VLs with NEUN+ neuronal cell bodies in the GZL *in GNG5* OX and 11% in controls) (Figure 1J). This difference was still significant at 35+7 days for MAP2 (33% of the *GNG5* OX VLs with MAP2+ neuronal cell bodies in the GZL and 5% in controls; 39% of the *GNG5* OX VLs with neuronal processes in the GZL and 33% in controls) (Figure 1G) and with a tendency for NEUN staining (18% of the VLs with NEUN+ neuronal cell bodies in the GZL *in GNG5* OX and 11% in controls) (Figure 1K). The presence of MAP2+ neuronal processes in the GZLs, especially during early developmental stages, reflects the proportion of migrating neurons that start to express markers of mature neurons. This proportion is naturally reduced at later time points as it was observed in control COs (Figures 1F,G,J,K). Interestingly, in *GNG5* OX COs the percentage of clear GZLs, without any process was not increased through time indicating a delay in neuronal migration (Figures 1F,G,J,K). Additionally, in more than 75% of the VLs at 20 + 7 days and more than 90% of VLs at 35+7 days the ectopic processes and ectopic cell bodies were GFP- indicating a cell non-autonomous role of GNG5 (highlighted in Figure 1C′,D′,E′). Both the altered radial morphology and delay in neuronal migration could be caused by premature delamination of progenitor cells, similarly to what was previously observed when *DCHS1* and *FAT4* are mutated (Klaus et al., 2019). Hence, since the delamination of RGs is accompanied by the loss of the apical junctions, we analyzed the integrity of the apical belt by PHALLODIN immunostaining, which labels the fraction of actin contained in fibers (F-ACTIN) (Figures 2A–C′). Missing PHALLOIDIN+ areas in the apical belt were found upon overexpression of *GNG5* at both stages analyzed. Around 40% of the VLs at 20 + 7 days and 27% of the VLs at 35 + 7 days showed an irregular apical belt compared to 11 and 9%, respectively in control COs (Figures 2D,E). To assess the possible cell non-autonomous role of GNG5, we divided the electroporated VLs into two categories, big or small electroporation, depending on if there were more than 20 GFP+ cells or not, respectively. Interestingly, the disruption of the apical belt was mainly found when the electroporation was big (82% of the electroporated VLs at 20 + 7 and 85% at 35 + 7 days) (Figures 2F,G). Taken together, these data suggest that forced expression of *GNG5* in developing COs induces morphological changes in electroporated RGs, leading to premature delamination. These alterations in the RGs may lead to failure in guiding migrating neurons to the CPL. Notably, these data are reminiscent of the phenotype observed in *DCHS1* and *FAT4* mutant COs in which RGs presented morphological alterations and consequently, neurons failed to migrate to the correct place. Since *GNG5* expression was strongly altered in *DCHS1* and *FAT4* mutant COs, we hypothesized that changes in *GNG5* expression may be responsible for the alterations found in those COs (Klaus et al., 2019), suggesting GNG5 as a key player in RG morphology and function.

### Forced Expression of *GNG5* Induces Alterations in RG Morphology and Cell Distribution *in vivo*

**FIGURE 3:**
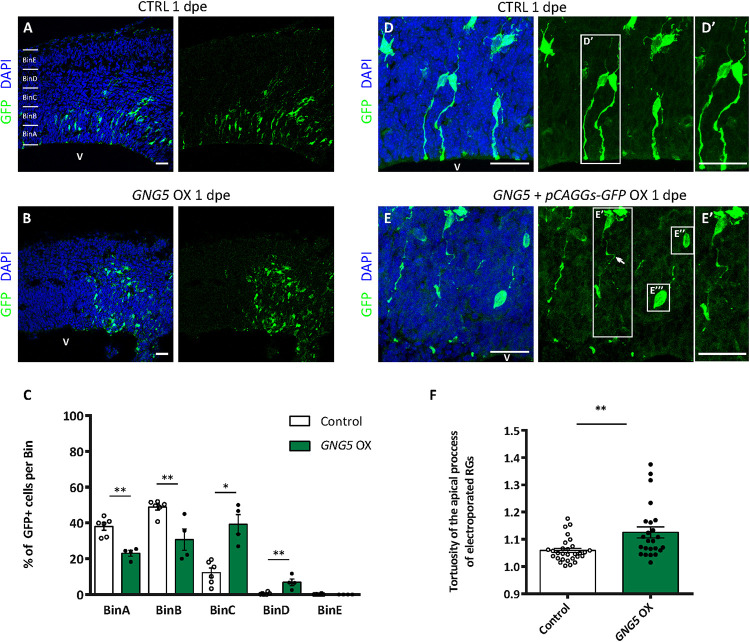
Acute overexpression of *GNG5* in mouse embryos at E13 alters transfected cell morphology and induces changes in the distribution of cells one day after electroporation. **(A,B)** Representative pictures of electroporated mouse brain sections stained for GFP. Sections were divided into five equal bins. **(A)** In embryos electroporated with the control plasmid, GFP+ cells stay in the first 2 bins while in **(B)** *GNG5* OX mice, GFP+ cells can be found in superficial bins. **(C)** Distribution of GFP+ cells per bin in control and *GNG5* OX mice at E13-E14. **(D–E″′)** Representative pictures of the morphology of transfected cells with control or *GNG5* plasmids co-electroporated with the control plasmid. (**E′**) Arrows indicate the cells with a non-straight morphology and (**E″,E″′**) cells that delaminate or do not have processes mainly present in *GNG5* OX embryos. (**F**) Tortuosity of the electroporated aRGs in control and *GNG5* OX mice. Statistical significance was based on Mann-Whitney test ^∗^*p* < 0.05 and ^∗∗^*p* < 0.01. **(A–C)** Control *n* = 6 and *GNG5* OX *n* = 4. (**D–F**) Control n (cells) = 29 and *GNG5* OX n (cells) = 24. Data are represented as mean ± SEM. Scale bar: 30 μm. Abbreviations: CTRL, control; OX, overexpression; V, ventricle.

To test whether GNG5 has a role during neurogenesis *in vivo*, we overexpressed *GNG5* by acute *in utero* electroporation in the developing mouse cortices at E13. Already at 1 dpe, we found differences in the position of the *GNG5* overexpressing cells, suggesting changes in their fate and/or migration (Figures 3A–C). To analyze in detail the position of electroporated cells, we subdivided the cortex into five equally distributed bins, spanning from the apical belt to the basement membrane. In control embryos, most of the GFP+ cells were distributed between BinA and BinB, the area approximately corresponding to the ventricular zone (VZ) and subventricular zone (SVZ), respectively (Figures 3A,C). On the contrary, in *GNG5* overexpressing embryos, GFP+ cells accumulated in BinB and BinC with a small fraction of cells in BinD (Figures 3B,C). Taking together the data obtained *in vitro* from COs and *in vivo* in the mouse cortex, we hypothesized that this misposition of the electroporated cells could be the result of premature delamination or premature differentiation of apical progenitor cells to intermediate progenitors (IPs) or neurons. To reveal if the increased number of GFP+ cells in BinC could be the result of premature differentiation, we quantified the number of Tbr2+ IPs. However, we did not find a significant difference in the proportion and the total number of Tbr2+ cells, but only a tendency of Tbr2+ cells accumulating in BinC and BinD at the expense of BinA and BinB (Supplementary Figures 3A–D).

In addition, to highlight the morphology of the cells we co-electroporated the embryos with the control *pCAGGS-GFP* plasmid only or with the *pCAGGS-GFP* together with the *pCAGGS-GFP-IRES-GNG5* (Figures 3D–F). Interestingly, upon *GNG5* OX, many of the GFP+ cells showed a different cell morphology (Figures 3D–E″′). Many of the cells did not have any processes (Figures 3E″,E″′) and from the ones that still had an aRGs-like structure the processes were less straight with a twisted morphology (Figures 3E,E′,F) suggesting that from early stages of development, *GNG5* OX induces morphological changes in the electroporated RGs.

### Overexpression of GNG5 Induces Proliferation and Alters the Proportion of Different Progenitor Types in vivo

**FIGURE 4:**
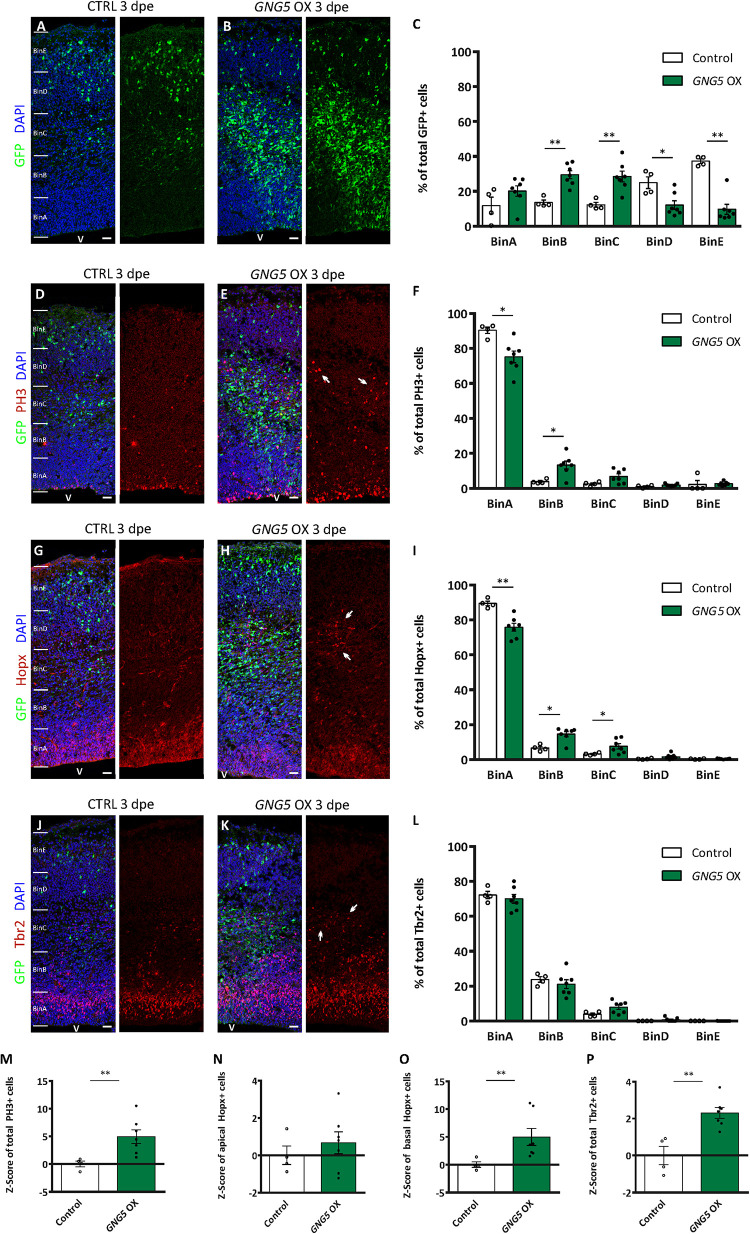
Acute overexpression of *GNG5* in mouse embryos at E13 alters the proportion of progenitors three days post electroporation. **(A,B)** Representative pictures of electroporated mouse brain sections stained for GFP and **(C)** distribution of GFP+ cells per bin. Sections were divided into five equal bins. In embryos electroporated with the control plasmid, GFP+ cells are distributed in all the bins while in *GNG5* OX mice, GFP+ cells can be found mainly in BinB and BinC. **(D,E)** Representative pictures of sections stained with PH3 and **(F)** distribution of PH3+ cells per bin. There is an increased amount of PH3 in *GNG5* OX in upper bins indicated by arrows. **(G,H)** Representative images of sections stained with Hopx and **(I)** distribution of Hopx+ cells per bin. In *GNG5* OX, there is an increased amount of Hopx+ cells in higher bins. Arrows indicate a cluster of Hopx+ cells. **(J,K)** Representative pictures of sections stained with Tbr2 and **(L)** distribution of Tbr2+ cells per bin. In *GNG5* OX, there is a general increase of Tbr2+ cells indicated with the arrows. The total number of cells per section are shown as Z-scores: **(M)** PH3+, **(N)** apical Hopx+, **(O)** basal Hopx+ and **(P)** Tbr2+. Statistical significance was based on Mann-Whitney test ^∗^*p* < 0.05 and ^∗∗^*p* < 0.01. Control *n* = 4 and *GNG5* OX *n* = 7. Data are represented as mean ± SEM. Scale bar: 30 μm. Abbreviations: CTRL, control; OX, overexpression; V, ventricle.

These minor but significant changes in the position of the electroporated cells, already visible *in vivo* a few hours after the overexpression of *GNG5*, prompted us to analyze the effect of overexpressing *GNG5* for a longer time in progenitor cells (Figure 4). 3 dpe (E13–E16), we found an increased number of GFP+ cells that accumulated in the intermediate zone (IZ) upon overexpression of *GNG5*. In contrast, in control embryos, GFP+ cells migrate to superficial bins (Figures 4A–C). To investigate the cellular identity of the cells that accumulated in the intermediate zone (BinC), we performed immunohistochemistry for the mitotic marker phosphohistone H3 (PH3). We found that the distribution of PH3+ was different between control and *GNG5* OX with increased accumulation of PH3+ cells in BinB at expenses of BinA in *GNG5* OX (Figures 4D–F). We also observed an increase in the total number of PH3+ progenitors in *GNG5* overexpressing cortices (Figure 4M). Interestingly, the increased number of PH3+ progenitors was mainly observed in GFP-cells, indicating (as in COs) a cell non-autonomous role of *GNG5*. Since *GNG5* is highly expressed in basal progenitors and especially in bRGs in humans and given that the majority of the GFP+ cells are found in BinB and C where basal progenitors are normally located, we wanted to investigate whether there were any changes in the number and distribution of these cells upon *GNG5* OX. Therefore, we performed immunohistochemical analysis using different basal progenitor markers. HOPX is enriched in human bRGs (Pollen et al., 2016) but in mice, Hopx, similarly to Pax6, is generally enriched in aRGs of the VZ since bRGs cells are rare (Wang et al., 2011; Telley et al., 2019; Figure 4G). Analysis of the number and distribution of Hopx+ cells upon *GNG5* OX showed an increased percentage of Hopx+ cells in BinB and BinC approximately corresponding to the SVZ and IZ, usually free of aRGs in the mouse brain (Figures 4G–I). Analysis of Pax6+ cells, also a marker of aRGs and bRGs, confirmed the presence of ectopic Pax6+ cells in the SVZ (BinB) and IZ (BinC) of the mouse embryos (Supplementary Figures 3H–I). Interestingly, the total number of apical Hopx+ cells (total number of Hopx+ cells in BinA and BinB) was not altered compared to control (Figure 4N) while the total number of basal Hopx+ cells (total number of Hopx+ cells in BinC-BinE) was different, indicating that the overexpression of *GNG5* leads to an increase of the number of RGs located in basal positions, above the VZ (Figure 4O). Besides, we observed a total increase in the number of Tbr2+ IPs (Figure 4P). We also analyzed the distribution of Tbr2+ IPs which was not altered between control and *GNG5* OX, due to their natural basal position (Figures 4J–L).

Altogether, these data show that overexpression of *GNG5* induces an increase in the proliferative capacity of the progenitor cells, especially of basally located progenitors which include IPs and bRGs. Our data also indicate that overexpression of *GNG5* can induce changes in the number of bRGs in mice. Strikingly, same as for PH3+, the ectopic Hopx+ and the increased number of Tbr2+ cells were GFP- which can indicate either a quick downregulation of the GFP after transfection or a cell non-autonomous role of *GNG5*. Finally, to compare the function of GNG5 in mouse and human progenitors, we analyzed the integrity of the apical belt by β-catenin immunohistochemistry. In most of the cases, the apical belt was similar to controls. However, two out of the seven embryos showed alterations of the apical membrane similar to the ones observed in COs, suggesting weaker penetrance compared to human RGs (Supplementary Figures 3E–G). Similar alterations in the distribution and amount of progenitor cells were previously observed upon downregulation of *Dchs1* and *Fat4* in mouse models, indicating that overexpression of *GNG5* and downregulation of *DCHS1* and *FAT4* induce similar phenotypes (Cappello et al., 2013).

### Overexpression of *GNG5* Affects Neuronal Distribution *in vivo*

**FIGURE 5:**
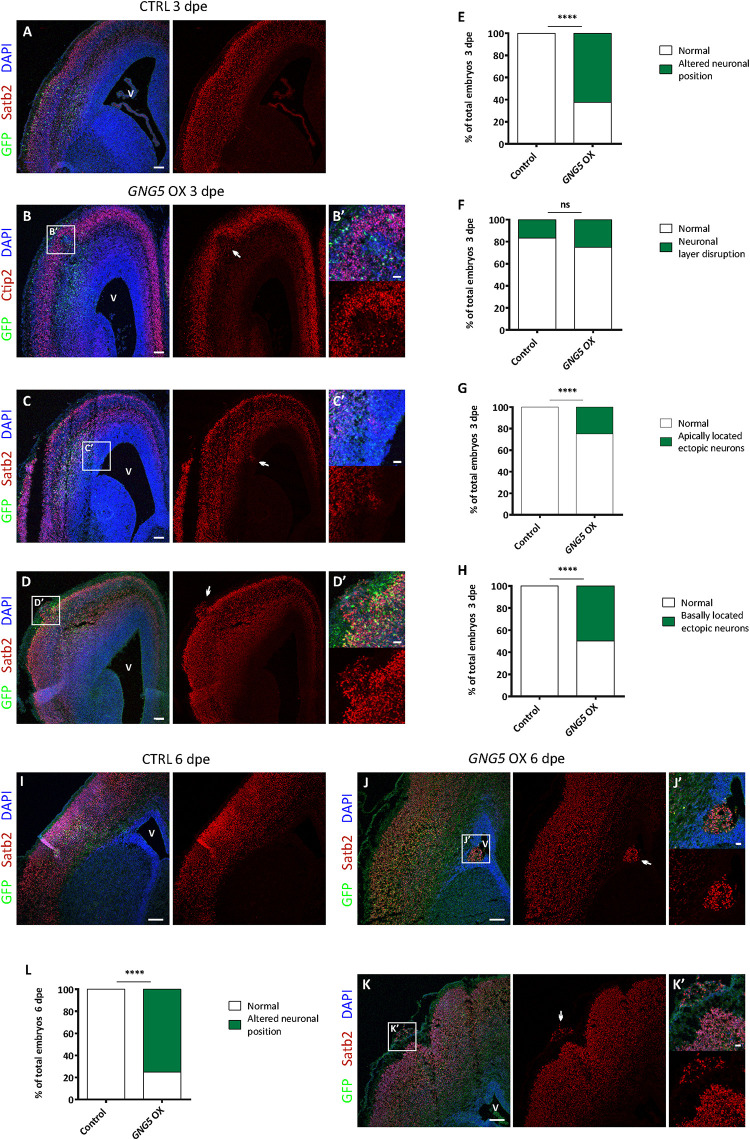
Acute overexpression of *GNG5* in E13 embryos induces migration defects in mice. (**A**) Representative images of a mouse section electroporated with a control plasmid at E13–E16 with an intact neuronal layer. **(B–D′)** Representative images of a brain section electroporated with the *GNG5* plasmid and showing different neuronal migration defects: **(B,B′)** neuronal layer disruption, **(C,C′)** apically located ectopic neurons or **(D,D′)** basally located ectopic neurons. **(E)** Quantification of the percentage of embryos with any of the neuronal migration defects at 3 dpe or **(F–H)** specific neuronal defects: **(F)** neuronal layer disruption, **(G)** apically located ectopic neurons or **(H)** basally located ectopic neurons. **(I)** Representative images of a mouse section electroporated with a control plasmid at E13-E19 with an intact neuronal layer. **(J–K′)** Representative images of brain sections electroporated with the *GNG5* plasmid and showing different neuronal migration defects: **(J,J′)** apically located ectopic neurons or **(K,K′)** basally located ectopic neurons. **(L)** Quantification of the percentage of embryos with neuronal migration defects at 6 dpe. Statistical analysis was based on exact binomial test ^****^p < 0.0001. Scale bar: **(B′–D′,J′, K′)** 30 μm, **(A–D)** 100 μm and (**I–K)** 150 μm. E13–E16 Control *n* = 6 and *GNG5* OX *n* = 8. E13–E19 Control *n* = 3 and *GNG5* OX *n* = 8. Abbreviations: CTRL, control; OX, overexpression; V, ventricle.

Increased numbers of progenitors at the basal location have been suggested to be one of the cellular steps for achieving the expansion and folding of the human cortex. Moreover, mutations in *DCHS1* and *FAT4* result in neurons with an altered transcriptional profile (high level of *GNG5*) and migratory dynamics. We, therefore, analyzed different neuronal markers upon forced expression of *GNG5* in mouse embryos electroporated at different developmental stages (3 and 6 dpe) (Figure 5).

Overexpression of *GNG5* interestingly resulted in neuronal phenotypes already at 3 dpe. *GNG5* overexpression led to different types of neuronal mispositioning (Figures 5A–D′) and the analysis showed that in most of the cases (62.5%) neuronal position alterations were present (Figure 5E). Specifically, 25% of embryos showed a disrupted neuronal layering (Figures 5A,B,B′,F), in 25% of embryos ectopic neurons were found at apical locations, resembling PH in human (Figures 5A,C,C′,G), and 50% of the embryos showed clusters of ectopic neurons at basal locations, sometimes resembling the formation of a small cobblestone (Figures 5A,D,D′,H). Analysis performed at later time points (6 dpe), confirmed similar, and even more pronounced neuronal defects. For instance, clusters of apically located ectopic neurons were more frequently found, as well as basally located ectopic neurons and rudimental folds (Figures 5I–K). Altogether, 75% of the embryos presented some type of immigration phenotype at this later stage (Figure 5L). Interestingly, the basement membrane was not completely compromised as shown by Laminin immunohistochemistry suggesting that its integrity was not the direct or indirect cause of the formation of cobblestones/folds (Supplementary Figure 4). Taken together, these data indicate that *GNG5* downregulation is critical for proper neuronal migration and neuronal layering during cortical development in mice.

## Discussion

In the present study, we investigated the role of GNG5 during cortical development and highlighted phenotypic similarities resulting from increased expression of *GNG5* and downregulation or mutations of *DCHS1* and *FAT4*, genes responsible for the presence of ectopic neurons in patients with Van Maldergem Syndrome.

On the one hand, the results of the study indicate that GNG5 may have a role in progenitor pool maintenance. Considering its high expression in early stages of development, especially in bRGs in humans (Supplementary Figures 1,2) and its regulation by well-known transcriptional factors important for neurogenesis such as PAX6 and SOX2 (Matys et al., 2003, 2006; Lachmann et al., 2010), this may not be surprising. Incessant expression of *GNG5* in progenitor cells after acute electroporation induces an increased proliferative capacity and amplified production of IPs as well as the presence of bRGs in mice. Basal progenitors and in particular bRGs, are highly enriched in the developing human cerebral cortex and are essential for the expansion and gyrification of the brain (Fietz et al., 2010; Kelava et al., 2012; Florio and Huttner, 2014; Penisson et al., 2019). Thus, our observation regarding the disruption in the neural layer and the increased number of basally located ectopic cells, which resemble the formation of cobblestone, may be explained by the increased production of bRGs in mice due to the acute overexpression of *GNG5*. Consequently, at later time points analyzed, basal clusters of neurons increase in size and the shape they acquire resembles the presence of folds.

On the other hand, force overexpression of *GNG5* also induces neuronal migration alterations leading to the presence of ectopic neurons in the germinal zones in mice and COs. The precise mechanism behind these migration defects remains elusive. The presence of a disrupted apical membrane in COs and to a lesser extent in mice could indicate premature delamination of aRGs which are the scaffold necessary for the neurons to reach their location in the cortex. Together with the altered morphology of the electroporated cells, these results could indicate that neurons may not be able to migrate properly due to an altered radial scaffold. We can, therefore, speculate that GNG5 is critical for the proliferation and differentiation of progenitors, and its downregulation is required for correct neuronal migration.

Similarly, in mice, acute downregulation of *Dchs1* and *Fat4* also induces an increased number of progenitors with a lower differentiation capacity resulting in ectopic neurons in the VZ (Cappello et al., 2013), while COs with mutations in *DCHS1* and *FAT4* present neuronal misposition of a specific subset of neurons and a disrupted morphology of aRGs (Klaus et al., 2019). Therefore, by mimicking the altered state of the subset of neurons in *DCHS1* and *FAT4* COs by overexpressing *GNG5*, we acquire similar results, suggesting that GNG5 could potentially mediate DCHS1 and FAT4 function. Rescue mediated by double knock-down of *GNG5* together with *DCHS1* or *FAT4* would suggest the convergent mechanism of these genes and similarities of phenotypes occurring upon upregulation of *GNG5* or downregulation of *DCHS1* and *FAT4*. However, we cannot exclude the role that the other dysregulated genes in *DCHS1* and *FAT4* mutant COs may have in proper proliferation, migration or axon guidance.

How DCHS1, FAT4 and GNG5 are interconnected is not known and the pathways by which GNG5 plays its role during cortical development remain elusive. However, we can, on the one side hypothesize how DCHS1, FAT4, and GNG5 may be linked and on the other side via which interactors GNG5 could function.

The subcellular localization of GNG5 in mitochondria correlates with the identification of the regulatory role of the atypical cadherin Fat in *Drosophila* (homologue of mammalian FAT4) in this cellular organelle (Sing et al., 2014). This data could indicate a role in the metabolism of these two proteins. Interestingly, it has also been shown that human-specific genes important for bRG proliferation such as *ARHGAP11B* are located in mitochondria (Namba et al., 2020). Moreover, it has been recently shown that cell fate decisions involve mitochondrial dynamics (Iwata et al., 2020).

The binding of GPCRs with their ligands allows the release of subunit βγ an to become effectors in a wide range of pathways (Gilman, 1987; McCudden et al., 2005). For instance, mutations in the G protein-coupled receptor 56 (GPR56 or ADGRG1), differentially expressed in *DCHS1* and *FAT4* mutant COs, result in cobblestones, caused by the disruption of the pial membrane and by an increase in the proliferative capacity of progenitor cells in mice (Li et al., 2008; Bae et al., 2014). In humans, mutations in this gene have been associated with bilateral frontoparietal polymicrogyria with phenotypic features of cobblestone-like lissencephaly (Bahi-Buisson et al., 2010) and one of the main characteristics is the presence of seizures. Endothelin receptor B (EDNRB) is another GPCR highly upregulated in the altered population of neurons in *DCHS1* and *FAT4* mutant COs (Klaus et al., 2019) and is important for the proliferation of neuronal progenitors in the cerebellum (Vidovic et al., 2008).

Finally, the cell non-autonomous role of GNG5 remains to be investigated. It has been demonstrated that GNG5, DCHS1, FAT4 and ADGRG1 are secreted and released in vesicles the size of exosomes (30–150 μm size) (Sharma et al., 2019). In consequence, all these proteins could be mediators of cell signaling and cell to cell crosstalk (Colombo et al., 2014) which is essential for correct neurogenesis (Sharma et al., 2019). The fact that all these proteins are present in exosomes could indicate a similar role in intercellular communication and that the defective levels or mutations of the secreted proteins could result in alterations of proper cortical development. Finally, it is possible that GNG5 is either secreted directly or that it may have a role in mediating the secretion of other proteins and these could explain the cell non-autonomous functions and its function during neurogenesis.

## Data Availability Statement

The original contributions presented in the study are included in the article/Supplementary Material, further inquiries can be directed to the corresponding author.

## Ethics Statement

Ethical review and approval was not required for the study on human participants in accordance with the local legislation and institutional requirements. Written informed consent for participation was not required for this study in accordance with the national legislation and the institutional requirements. The animal study was reviewed and approved by license number 55.2-1-54-2532-79-2016 approved by the Government of Upper Bavaria.

## Author Contributions

SC and ACA-M: conceptualization and investigation. ACA-M, CK, and RD: methodology. SC: resources, writing – review and editing, visualization, supervision, and funding acquisition. ACA-M: writing – original draft. All authors contributed to the article and approved the submitted version.

## Conflict of Interest

The authors declare that the research was conducted in the absence of any commercial or financial relationships that could be construed as a potential conflict of interest.

## Abbreviations

aRG: apical radial glial cells
bRG: basal radial glial cell
COs: cerebral organoids
CPL: cortical plate-like area
CTRL: control
DPE: day post electroporation
EX: Embryonic day X
GPCR: G-coupled receptors
GZL: germinal zone-like area
IP: intermediate progenitor
iPSC: induced pluripotent stem cells
IZ: intermediate zone
OX: overexpression
PH: periventricular heterotopia
PH3: phosphohistone H3
RG: radial glial cell
RT: room temperature
SVZ: subventricular zone
V: ventricle
VL: ventricle-like cavities
VZ: ventricular zone.
