# Supplementary material for: GNG5 Controls the Number of Apical and Basal Progenitors and Alters Neuronal Migration During Cortical Development
Source: Front Mol Biosci. 2020 Nov 2;7:578137. doi: 10.3389/fmolb.2020.578137 (PMC7673377; doi:10.3389/fmolb.2020.578137)
Supplement: Supplementary file 1 [file Data_Sheet_1.pdf]

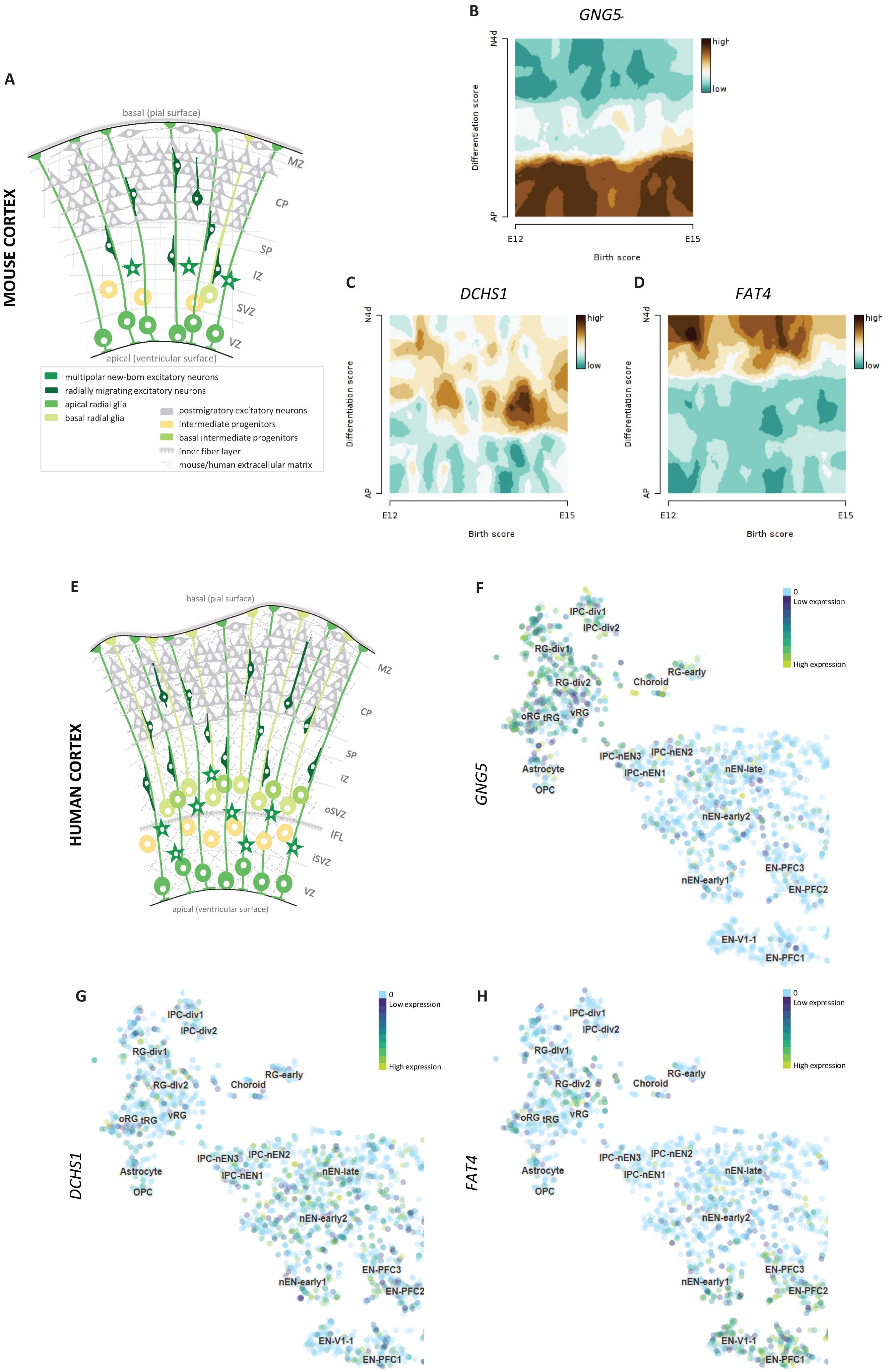

A
MOUSE CORTEX
basal (pial surface)
MZ
CP
SP
IZ
SVZ
VZ
apical (ventricular surface)
multipolar new-born excitatory neurons
radially migrating excitatory neurons
apical radial glia
basal radial glia
postmigratory excitatory neurons
intermediate progenitors
basal intermediate progenitors
inner fiber layer
mouse/human extracellular matrix
B
GNG5
N4d
Differentiation score
AP
E12
E15
Birth score
high
low
C
DCHS1
D
FAT4
E
HUMAN CORTEX
oSVZ
IFL
iSVZ
F
GNG5
0
Low expression
High expression
IPC-div1
IPC-div2
RG-div1
RG-div2
RG-early
Choroid
oRG
tRG
vRG
Astrocyte
OPC
IPC-nEN3
IPC-nEN2
IPC-nEN1
nEN-late
nEN-early2
nEN-early1
EN-PFC3
EN-PFC2
EN-V1-1
EN-PFC1
G
DCHS1
H
FAT4

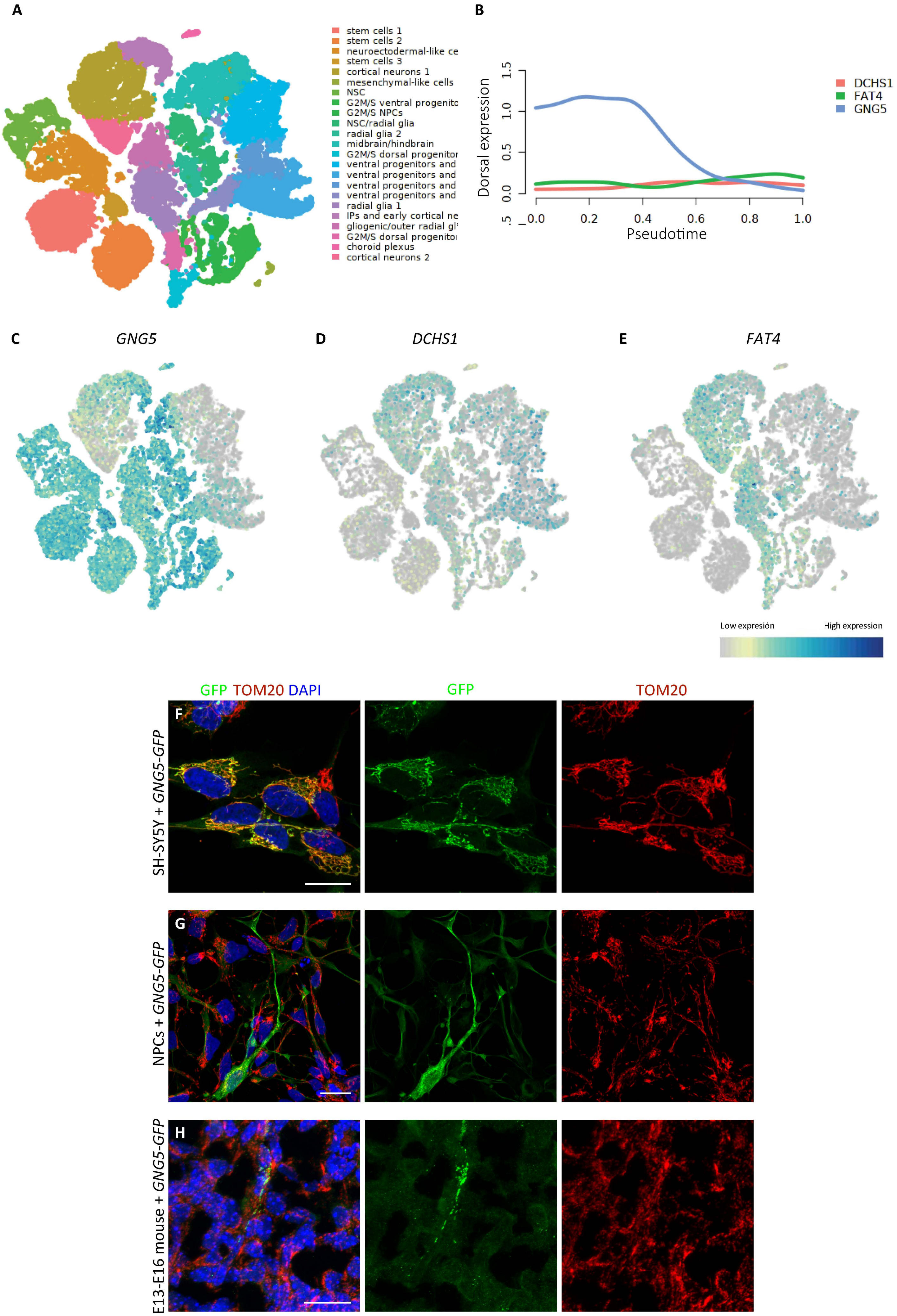
A
stem cells 1
stem cells 2
neuroectodermal-like ce
stem cells 3
cortical neurons 1
mesenchymal-like cells
NSC
G2M/S ventral progenitc
G2M/S NPCs
NSC/radial glia
radial glia 2
midbrain/hindbrain
G2M/S dorsal progenitor
ventral progenitors and
ventral progenitors and
ventral progenitors and
ventral progenitors and
radial glia 1
IPs and early cortical ne
gliogenic/outer radial gl
G2M/S dorsal progenitor
choroid plexus
cortical neurons 2
B
Dorsal expression
0.0
0.5
1.0
1.5
0.0
0.2
0.4
0.6
0.8
1.0
Pseudotime
DCHS1
FAT4
GNG5
C
GNG5
D
DCHS1
E
FAT4
Low expresión
High expression
GFP TOM20 DAPI
GFP
TOM20
F
SH-SY5Y + GNG5-GFP
G
NPCs + GNG5-GFP
H
E13-E16 mouse + GNG5-GFP

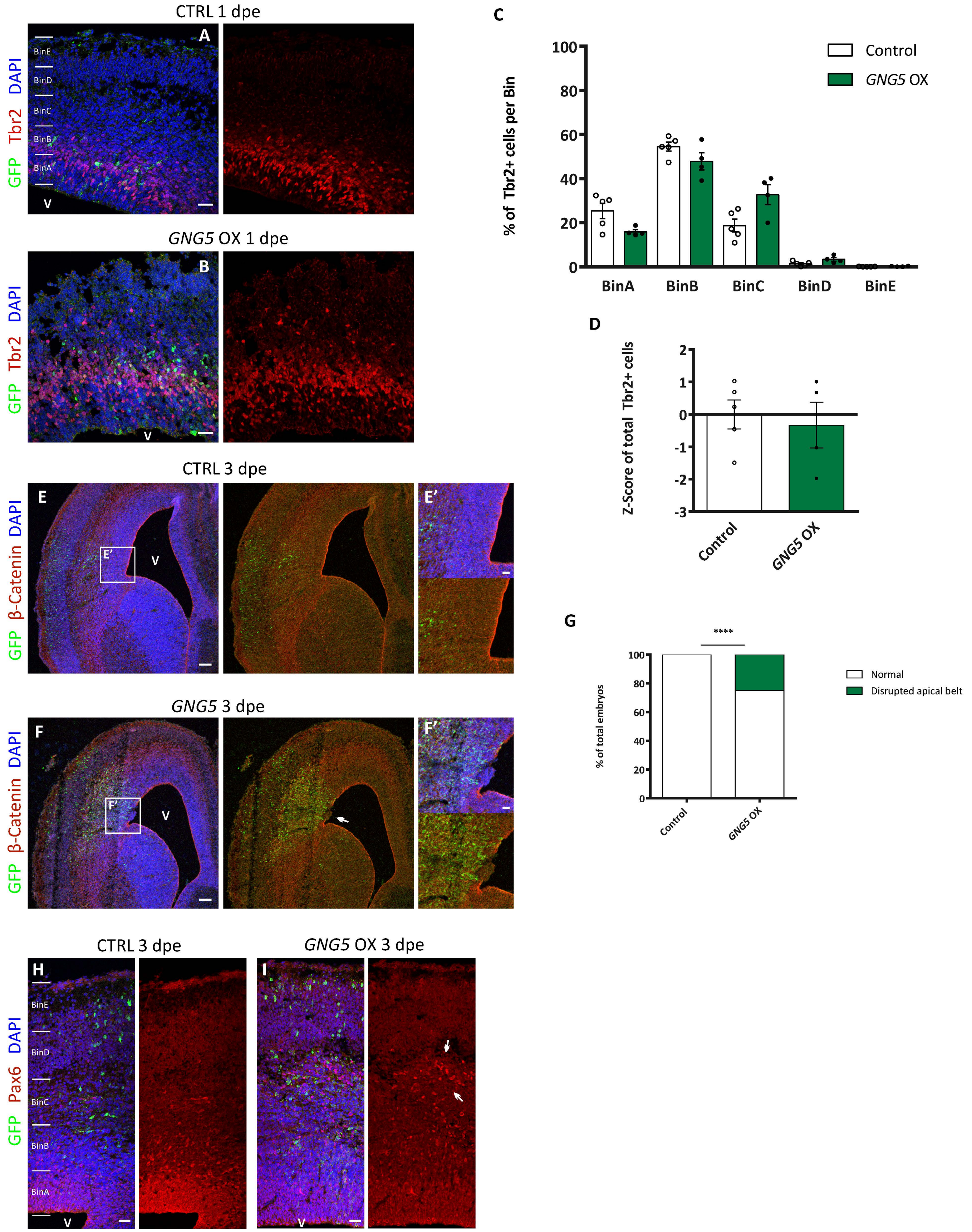
CTRL 1 dpe
A
BinE
BinD
BinC
BinB
BinA
V
GFP Tbr2 DAPI
GNG5 OX 1 dpe
B
V
GFP Tbr2 DAPI
C
% of Tbr2+ cells per Bin
100
80
60
40
20
0
BinA
BinB
BinC
BinD
BinE
Control
GNG5 OX
D
Z-Score of total Tbr2+ cells
2
1
0
-1
-2
-3
Control
GNG5 OX
CTRL 3 dpe
E
E'
V
GFP β-Catenin DAPI
GNG5 3 dpe
F
F'
V
GFP β-Catenin DAPI
G
****
% of total embryos
100
80
60
40
20
0
Control
GNG5 OX
Normal
Disrupted apical belt
CTRL 3 dpe
H
BinE
BinD
BinC
BinB
BinA
V
GFP Pax6 DAPI
GNG5 OX 3 dpe
I
V

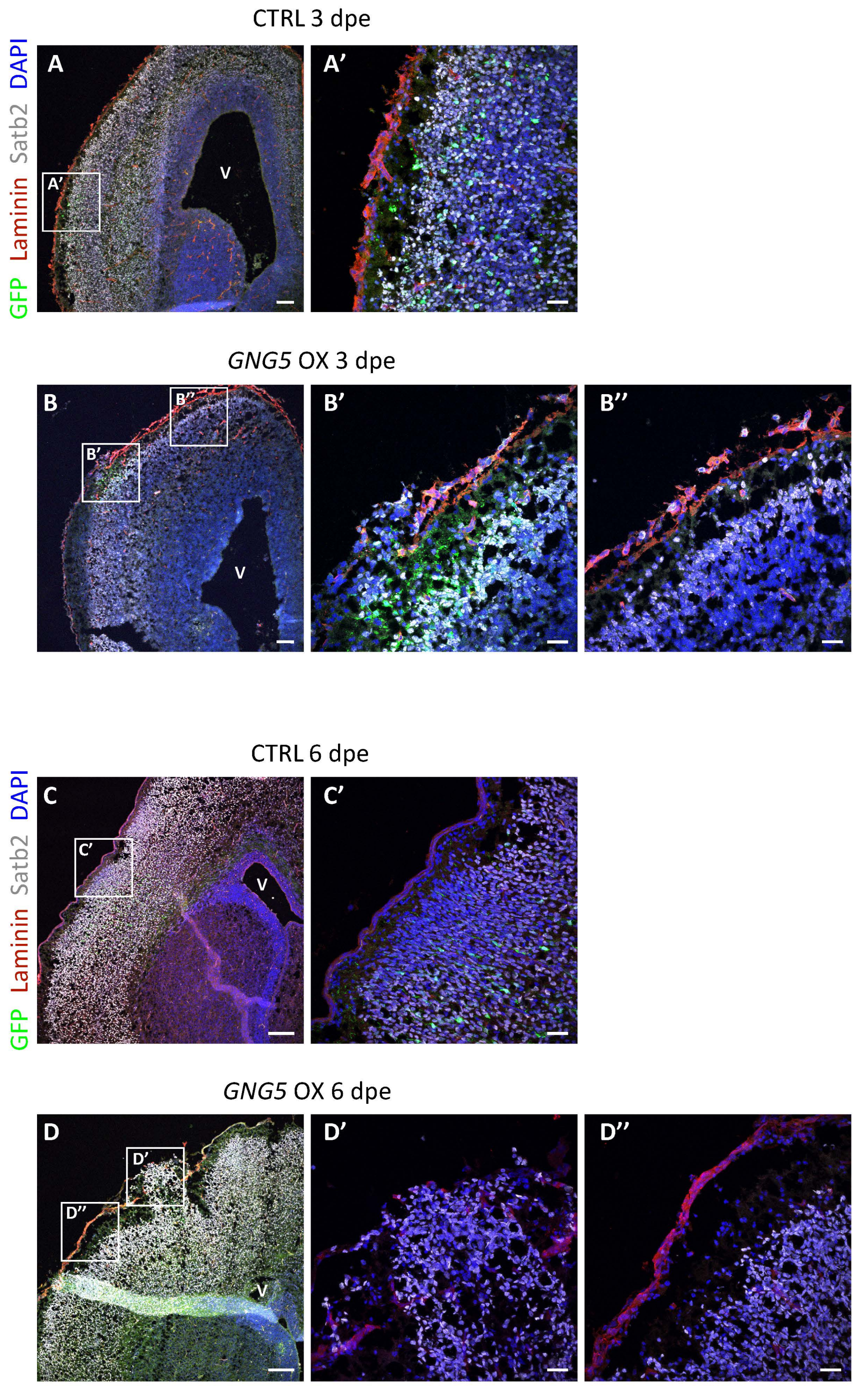
CTRL 3 dpe
GFP Laminin Satb2 DAPI
A
A'
V
GNG5 OX 3 dpe
B
B'
B''
V
CTRL 6 dpe
GFP Laminin Satb2 DAPI
C
C'
V
GNG5 OX 6 dpe
D
D'
D''
V

Supplementary Figure 1: *Gng5*, *Dchs1* and *Fat4* expression levels in mice and humans over time in different cell types.

(**A**) Schematic of the mouse developing cortex. **(B-D)** Transcriptional landscape of (**B**) *Gng5* (**C**) *Dchs1* and (**D**) *Fat4* expression in the developing mouse cortex. The heat-maps show the expression of the genes in the different cell clusters of cells (Ap→N4d) over time (E12→E15) (Telley et al., 2019). While *Dchs1* and *Fat4* share a similar expression pattern and are mainly expressed in neurons, *Gng5* expression is stable over time with its higher expression in apical cells. (**E**) Schematic of the human developing cortex. **(F-G)** Cell-type expression of each gene over time (age is represented in weeks). (**F**) *GNG5*, (**G**) *DCHS1* and (**H**) *FAT4* in the different cell clusters over time. While *DCHS1* and *FAT4* expression are scarcer with a higher level in neuronal cells, *GNG5* expression localized mainly to progenitor cells with higher expression in RG, IPC and oRGs. Single-cell sequencing data from (Nowakowski et al., 2017). Abbreviations: (mouse data), Ap: apical progenitors, EX: embryonic day, N4d: 4-day-old neurons and (human data), IPC: intermediate progenitor, IPC-div: intermediate progenitor dividing, IPC-nEN: intermediate progenitors-early born excitatory neuron, nEN: newborn excitatory neuron, RG: radial glia, RG-div: radial glia dividing, OCP: oligodendrocyte progenitor, oRGs: outer radial glia, vRG: ventral radial glia.

Supplementary Figure 2: *GNG5, DCHS1* and *FAT4* expression levels in human-derived COs over time in different cells types and cellular localization of *GNG5* in different cell types.

(**A**) Legend for the single-cell RNA sequencing data from human-derived COs (Kanton et al., 2019). (**B**) Temporal expression of *GNG5*, *DCHS1*, and *FAT4* over time. While *DCHS1* and *FAT4* expressions start later in time, *GNG5* is mainly expressed at the beginning of cortical development. (**C-E**) Expression levels of (**C**) *GNG5*, (**D**) *DCHS1* and (**E**) *FAT4* in the different cell clusters in COs. While *DCHS1* and *FAT4* are mainly expressed in neuronal cells *GNG5* expression is higher in progenitor cells. (**F-H**) Mitochondrial localization of GNG5 in neuroblastoma (SHSY-5Y), human NPCs and in embryonic mouse cortex (electroporated at E13 and analyzed 3dpe). Scale bar 20 µm. Abbreviations: IP: intermediate progenitor, NPC: neural precursor cell, NSC: neural stem cell.

Supplementary Figure 3: Acute overexpression of *GNG5* induces alteration in the mouse developing cortex.

(A, B) Representative pictures of electroporated mouse brain sections electroporated at E13 and analyzed 1 dpe with Tbr2 staining. Sections were divided into five bins. **(C)** Distribution of Tbr2+ cells per bin and **(D)** the total number of cells per section shown as Z-scores. There is no significant difference in the distribution between control and *GNG5* OX mice neither in the total amount of Tbr2+ cells. **(E-F`')** Representative pictures of mouse embryos transfected at E13 and analyzed 3 dpe stained for β-Catenin to look at the integrity of the apical belt. **(G)** Quantification of the percentages of animals with apical membrane integrity defects. *GNG5* OX mice present a higher number of embryos with a disrupted apical belt. **(H-I)** Representative pictures of electroporated mouse brain sections at E13 and stained with Pax6 3 dpe. Arrows indicate the presence of ectopic Pax6 cells in the *GNG5* OX brains. For Tbr2 analysis statistical significance was based on Mann-Whitney test and for β-Catenin statistical analysis was based on exact binomial test ****p < 0.0001. Tbr2: Control n=5 and *GNG5* OX n=4. β-Catenin: Control n=6 and

GNG5 OX *n*=8. Data are represented as mean ± SEM**.** Scale bar: **(A, B, E', F', H, I)** 30 µm and **(E, F)** 100 µm. Abbreviations: CTRL: control, OX: overexpression, V: ventricle.

Supplementary Figure 4: Acute overexpression of *GNG5* induces migration defects in mice.
Representative pictures of electroporated mouse brain sections electroporated at E13 and analyzed 3 dpe and 6 dpe. **(A, A')** Staining for Laminin indicates the intact basal membrane in control embryos at E13-E16. **(B, B', B'')**. Overexpression of *GNG5* induces the accumulation of basally located ectopic neurons. The basal membrane stays intact in E13-E16. **(C, C')** Intact basal membrane in control E13-E19 embryos and **(D, D', D'')** slightly altered membrane in *GNG5* OX embryos at E13-E19. Scale bar: **(A', B', B'', C', D', D'')** 30 µm, **(A, B)** 100 µm and **(C, D)** 150 µm. Abbreviations: CTRL: control, OX: overexpression, V: ventricle.

References

Kanton, S., Boyle, M. J., He, Z., Santel, M., Weigert, A., Sanchís-Calleja, F., et al. (2019). *Organoid single-cell genomic atlas uncovers human-specific features of brain development*. doi:10.1038/s41586-019-1654-9.

Nowakowski, T. J., Bhaduri, A., Pollen, A. A., Alvarado, B., Mostajo-Radji, M. A., Di Lullo, E., et al. (2017). Spatiotemporal gene expression trajectories reveal developmental hierarchies of the human cortex. *Science* 358, 1318–1323. doi:10.1126/science.aap8809.

Telley, L., Agirman, G., Prados, J., Amberg, N., Fièvre, S., Oberst, P., et al. (2019). Temporal patterning of apical progenitors and their daughter neurons in the developing neocortex. *Science* 364. doi:10.1126/science.aav2522.
